# Meiotic Behavior of Achiasmate Sex Chromosomes in the African Pygmy Mouse *Mus mattheyi* Offers New Insights into the Evolution of Sex Chromosome Pairing and Segregation in Mammals

**DOI:** 10.3390/genes12091434

**Published:** 2021-09-17

**Authors:** Ana Gil-Fernández, Marta Ribagorda, Marta Martín-Ruiz, Pablo López-Jiménez, Tamara Laguna, Rocío Gómez, María Teresa Parra, Alberto Viera, Frederic Veyrunes, Jesús Page

**Affiliations:** 1 Departamento de Biología, Facultad de Ciencias, Universidad Autónoma de Madrid, 28049 Madrid, Spain; anula.ulula@gmail.com (A.G.-F.); martariba2@gmail.com (M.R.); ruizm5@upmc.edu (M.M.-R.); pablo.lopezj@uam.es (P.L.-J.); tamara.laguna@estudiante.uam.es (T.L.); rocio.gomez@uam.es (R.G.); mayte.parra@uam.es (M.T.P.); alberto.viera@uam.es (A.V.); 2 Institut des Sciences de l’Evolution, ISEM UMR 5554 (CNRS/Université Montpellier/IRD/EPHE), 34090 Montpellier, France; frederic.veyrunes@umontpellier.fr

**Keywords:** sex chromosomes, meiosis, evolution, pygmy mouse, *Mus mattheyi*

## Abstract

X and Y chromosomes in mammals are different in size and gene content due to an evolutionary process of differentiation and degeneration of the Y chromosome. Nevertheless, these chromosomes usually share a small region of homology, the pseudoautosomal region (PAR), which allows them to perform a partial synapsis and undergo reciprocal recombination during meiosis, which ensures their segregation. However, in some mammalian species the PAR has been lost, which challenges the pairing and segregation of sex chromosomes in meiosis. The African pygmy mouse *Mus mattheyi* shows completely differentiated sex chromosomes, representing an uncommon evolutionary situation among mouse species. We have performed a detailed analysis of the location of proteins involved in synaptonemal complex assembly (SYCP3), recombination (RPA, RAD51 and MLH1) and sex chromosome inactivation (γH2AX) in this species. We found that neither synapsis nor chiasmata are found between sex chromosomes and their pairing is notably delayed compared to autosomes. Interestingly, the Y chromosome only incorporates RPA and RAD51 in a reduced fraction of spermatocytes, indicating a particular DNA repair dynamic on this chromosome. The analysis of segregation revealed that sex chromosomes are associated until metaphase-I just by a chromatin contact. Unexpectedly, both sex chromosomes remain labelled with γH2AX during first meiotic division. This chromatin contact is probably enough to maintain sex chromosome association up to anaphase-I and, therefore, could be relevant to ensure their reductional segregation. The results presented suggest that the regulation of both DNA repair and epigenetic modifications in the sex chromosomes can have a great impact on the divergence of sex chromosomes and their proper transmission, widening our understanding on the relationship between meiosis and the evolution of sex chromosomes in mammals.

## 1. Introduction

Meiosis is a specialized type of cell division essential for the transmission of chromosomes across generations [1]. During prophase-I, homologous chromosomes pair and associate all along their length owing to the assembly of a specific structure, the synaptonemal complex (SC), that holds the two homologs together [2,3]. Concomitantly, homologous chromosomes undergo recombination. This is a DNA repair process that initiates at the beginning of meiosis with the endogenous production and processing of hundreds of DNA double strand breaks (DSBs) by SPO11 (a topoisomerase-like protein) and a number of associated proteins, including the MRN (MRE11-RAD51-NBS1) and RMM (REC114-MER2-MEI4) complexes [4,5]. The production of DSBs triggers a DNA repair response through the homologous recombination pathway. DNA end resection following DSB events produces single stranded DNA overhangs that become protected by RPA protein. This is subsequently replaced by recombinases RAD51 and DMC1, which promote the interaction and recognition of homologous chromosomes and, hence, chromosome synapsis. Some of these DSBs are repaired taking the homologous chromosomes as a template, which can eventually lead to the formation of crossovers, whose cytological manifestation are chiasmata. The latter are essential for maintaining the association of homologous chromosomes until the metaphase-I/anaphase-I transition, when these chromosomes segregate to opposite cell poles. Therefore, the proper segregation of homologous chromosomes during first meiotic division depends on their recognition and association in prophase-I through a homology-based mechanism [6,7].

While most chromosomes follow this general pattern, sex chromosomes in mammalian male meiosis are prone to present exceptions to some of these processes. In most species the X and Y chromosomes are highly differentiated in size and gene content [8,9]. Nevertheless, they still share a region of homology called pseudo-autosomal region (PAR) [10]. In this short region sex chromosomes synapse, recombine and form a chiasma that ensures their proper segregation [11,12,13,14]. However, the differences in size and gene content introduce dramatic changes in the meiotic behavior of sex chromosomes. First, a large portion of the X and the Y chromosomes remains unsynapsed [13,15,16]. This fact triggers a mechanism of transcription inactivation called meiotic sex chromosome inactivation (MSCI), which involves the deposition of a number of epigenetic factors on the sex chromosomes and the formation of a compact chromatin mass (the sex body) [17,18,19,20]. Second, the production of DSBs in the PAR is delayed and highly regulated. In the house mouse *Mus musculus,* it was reported that these DSBs are introduced by the SPO11α isoform, acting only in the PAR at the end of zygotene [21,22]. According to this, usually a single DSB focus is observed in the Y chromosome, appearing at late zygotene [14,21]. The production of this DSB requires in addition the accumulation in the PAR of many recombination related proteins, including REC114, MEI4, MEI1, ANKRD31 and IHO1, which form the so called RMMAI complex [23], and from which ANKRD31 seems critical [24,25]. Finally, it has been proposed that DNA repair in the non-homologous region of sex chromosomes would occur through homologous recombination, but using the sister chromatid as template, since a homologous chromosome is not available [22,26,27,28].

Although the presence of the PAR is widespread in mammals, some species present completely differentiated sex chromosomes (i.e., the PAR has been lost) [29,30,31,32,33,34,35,36]. This situation challenges the regular mechanisms to ensure a proper transmission of these chromosomes, since in the absence of synapsis and recombination sex chromosomes become achiasmate. Previous studies have revealed that different mammalian groups have developed different strategies to overcome this problem. In marsupials, sex chromosomes are associated during prophase I by a proteinaceous structure, the dense plate, which develops over the internal surface of the nuclear envelope at pachytene and maintains sex chromosome association until they segregate at anaphase-I [37,38,39,40,41,42,43,44]. This structure includes some of the components of the SC, most relevantly the SYCP3 protein [38]. Interestingly, a similar mechanism has been found in distantly related mammals, such as gerbils and voles, in which SYCP3 forms filaments or aggregates that maintain the association of sex chromosomes until anaphase-I [45,46]. This implies that the SC components have been used recurrently and independently in different mammalian groups to cope with achiasmate sex chromosomes. Moreover, telomere or heterochromatin associations have also been related with the segregation of achiasmate sex chromosomes [29,33,34].

In order to gain insights into the meiotic behavior of achiasmate sex chromosomes, here we have studied male meiosis in the African pygmy mouse *Mus mattheyi*. The genus *Mus* is composed of a large number of species that are very similar phenotypically, but show great genetic divergence [47]. African pygmy mice are a group of small rodents (ranging from 4 to 15 g) included in the subgenus *Nannomys* [48,49], which are distributed throughout the African sub-Saharan zone. This subgenus is characterized by a great karyotypic diversity mainly due to autosomal centric fusions, but also to sex-autosome fusions. Thus, diploid numbers that range from 2*n* = 16 to 2*n* = 36 [50]. *M. mattheyi* [51] is one of the 18 species of the subgenus *Nannomys*, with an ancestral-like 2*n* = 36 karyotype where all chromosomes are acrocentric, meaning no centric fusions between autosomes nor between sex chromosomes and autosomes [52]. It was proposed that the X and Y chromosomes of the pygmy mouse species would not share any region of homology and, therefore, could be achiasmate during male meiosis [53,54,55,56,57], but a detailed analysis is lacking. Meiosis has been studied only in another pygmy mouse species, *Mus minutoides*, which differs to other pygmy mice because in this species a sex-autosome fusion between both the X and the Y and the autosomal pair 1 have restored a large neo-PAR that synapses and recombines at meiosis [55,57]. Therefore, here we have characterized for the first time the meiotic behavior of fully achiasmate sex chromosomes in this group. We have analyzed the localization of proteins involved in SC formation, the recombination process, and MSCI during male meiosis. Our results indicate that sex chromosome pairing is delayed in this species and there is not true synapsis. In addition, the regulation of DSB production and repair in the Y chromosome must be distinct since the dynamics of some DNA repair proteins does not coincide with that observed in other mouse species. Finally, we found that segregation of X and Y chromosome relies on chromatin connections involving some epigenetic modifications, like the phosphorylation of histone H2AX (γH2AX). Overall, the results obtained in this species suggest a key role of meiotic regulation on the evolution of sex chromosomes.

## 2. Material and Methods

### 2.1. Animals

*M. mattheyi* were bred in captivity at the CECEMA facilities of Montpellier University. The colony was established and maintained under standard conditions as previously reported [58]. Males were sacrificed by cervical dislocation and their testes processed for immunocytology. All experiments were conducted according to ethical rules established by the Institut des Sciences de l’Evolution of Montpellier and the Universidad Autónoma de Madrid (Ethics Committee Certificate CEI 55-999-A045).

### 2.2. Immunofluorescence

We prepared spermatocyte spreads and squashes following the procedures previously described [59,60]. Slides were incubated with primary antibodies diluted in phosphate buffered saline (PBS): 137 mM NaCl, 2.7 mM KCl, 10.1 mM Na_2_HPO_4_, 1.7 mM KH_2_PO_4_, pH 7.4) overnight at room temperature in a humid chamber. The following primary antibodies and dilutions used were: rabbit anti SYCP1 (Abcam 15090, Cambridge, UK), 1:100; rabbit anti SYCP3 (Abcam 15093), 1:100; mouse anti SYCP3 (Abcam 97672), 1:100; mouse anti γH2AX (Upstate #05-636, Millipore, Burlington, MA, USA), 1:1000; rabbit anti histone H2AX phosphorylated at serine 139 (γH2AX) (Abcam 2893), 1:1000; rabbit anti RPA2 (Abcam 10359), 1:100; rabbit anti RAD51 (CalBiochem PC-130, Millipore, Burlington, MA, USA), 1:50; mouse anti-MLH1 (Pharmingen 550838, BD Biosciences, Franklin Lakes, NJ, USA), 1:100; and a human serum that recognizes centromere proteins (#15-235, Antibodies Inc., Davis, CA, USA), 1:100. After rinsing in PBS, the slides were incubated with secondary antibodies diluted to 1:100 in PBS for one hour at room temperature: donkey anti-mouse, donkey anti rabbit and goat anti-human, conjugated with Alexa 350, Alexa 488, Alexa 549 (Invitrogen, Eugene, OR, USA), Cy3 or Dylight 649 (Jackson ImmunoResearch Laboratories, West Grove, PA, USA). Slides were counterstained with DAPI, when needed, and mounted with Vectashield (Vector, Burlingame, CA, USA).

Observations were made on an Olympus BX61 microscope equipped with a motorized plate in the *Z* axis. Images were captured with an Olympus DP61 camera and processed with Adobe Photoshop CS software. The length of the SC was measured using ImageJ software. For the squashes, several optical sections were recorded for each cell. The stack files were processed with ImageJ to make three-dimensional reconstructions as previously described [43,45].

## 3. Results

**Figure 1:**
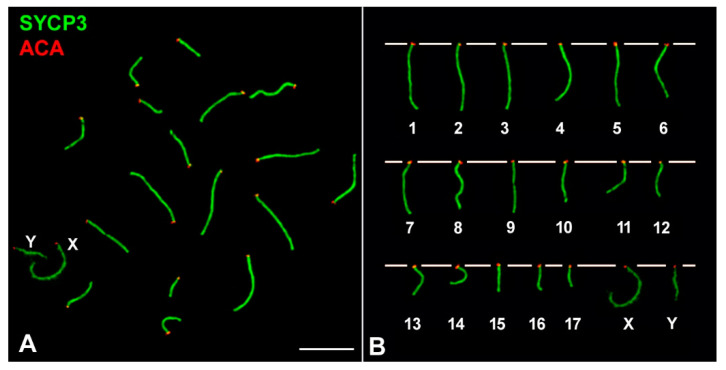
Chromosomes of *Mus mattheyi* (**A**) Spread spermatocyte at pachytene labelled with antibodies against SYCP3 (green) and centromeres (red). (**B**) Meiotic karyotype. Bivalents are arranged according to their length. The sex chromosomes (X, Y) are indicated. Scale bar: 10 µm.

Males of *M. mattheyi* have a meiotic karyotype of 2*n* = 36, with 17 autosomal bivalents plus the X and Y sex chromosomes, all of them being acrocentric (Figure 1). The X is the largest chromosome, representing 9.63% (±0.97) of the total SC length in pachytene spermatocytes (*n* = 11 spermatocytes), while the Y chromosome is small and represents 4.35% (±0.70) of the total SC.

**Figure 2:**
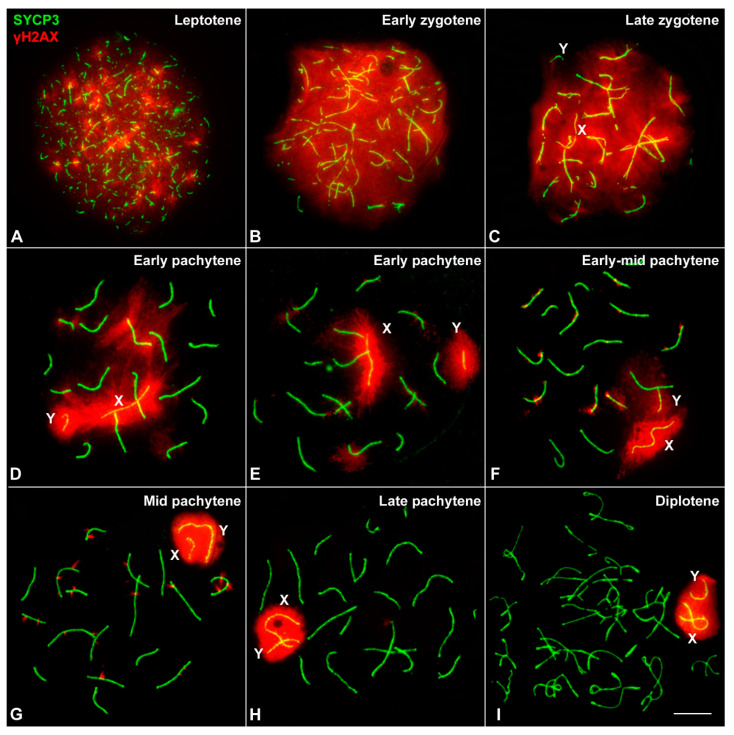
Prophase I progression in *M. mattheyi* spermatocytes. SYCP3 (green) and γH2AX (red). (**A**) Leptotene. Axial elements (AEs) appear as short filaments and γH2AX labelling appears as large foci scattered over the nucleus. (**B**) Early zygotene. AEs associate in some regions. γH2AX signal occupies the entire nucleus. (**C**) Late zygotene. Most autosomes have completed synapsis. γH2AX labelling still covers most of the nucleus. Sex chromosomes (X, Y) are distinguishable from the rest of chromosomes as thinner filaments. The Y chromosome has a single focus of γH2AX while the X is intensely labelled. (**D**) Early pachytene. All autosomes have completed synapsis, but large γH2AX foci remain associated with many of them. The AEs of sex chromosomes remain apart and surrounded by a large γH2AX signal. (**E**) Early pachytene. Sex chromosomes are completely separated from each other. An intense and irregular γH2AX signal surrounds each sex chromosome. (**F**) Early-mid pachytene. γH2AX signal in the autosomes appears as small foci closely associated to the SCs. The AE of the X chromosome is bent and the signal of γH2AX is more concentrated around the AE in both the X and the Y chromosomes. (**G**) Mid pachytene. γH2AX remains in many autosomes as small foci. Sex chromosomes appear closely associated inside a single γH2AX signal, which shows a compact appearance and a well-defined outline. No contact between their AEs is observed. (**H**) Late pachytene. Sex chromosomes remain associated but without contact of their AEs. γH2AX is only observed in the sex body. (**I**) Diplotene. Autosomes desynapse and remain associated only in some regions. Sex chromosomes remain associated inside a sex body intensely labelled with γH2AX. Scale bar: 10 µm.

The progression of prophase-I was characterized through the immunolocalization of SYCP3, a component of the axial-lateral elements (AEs/LEs) of the SC, and γH2AX (histone H2AX phosphorylated at serine 139), a histone modification that marks chromosomal regions that present DNA damage and/or have not completed synapsis and is also a typical marker of MSCI. The localization and morphological pattern of these two proteins allow the precise discrimination of the different stages in mammalian meiosis [27]. In leptotene, AEs of chromosomes start to form short and discontinuous filaments labelled with SYCP3, while γH2AX appears as large foci that cover almost the whole nucleus, indicating the induction of DNA damage (Figure 2A). At early zygotene, chromosome AEs are almost completely formed as continuous filaments, homologous chromosomes begin to synapse and γH2AX is homogeneously distributed over the nucleus (Figure 2B). At late zygotene (Figure 2C), synapsis has progressed extensively in all chromosomes and γH2AX signal begins to be lost in some regions of the nucleus, but its labelling is still quite widespread. Synapsis between homologues is completed at pachytene. It is possible to distinguish different sub-stages during pachytene owing to differences in the distribution of γH2AX. At early pachytene (Figure 2D), γH2AX is still detectable as a cloud of considerable extension and intensity around the SC of some autosomes, despite having completed synapsis, and on the sex chromosomes, which appear conspicuously labelled from this stage onwards (Figure 2D–I). At mid pachytene (Figure 2G), γH2AX is observed as discrete and small foci associated to the SC of autosomes. These foci are not detected at late pachytene (Figure 2H) and diplotene, when homologous chromosomes desynapse (Figure 2I).

### 3.1. Sex Chromosome Pairing

**Figure 3:**
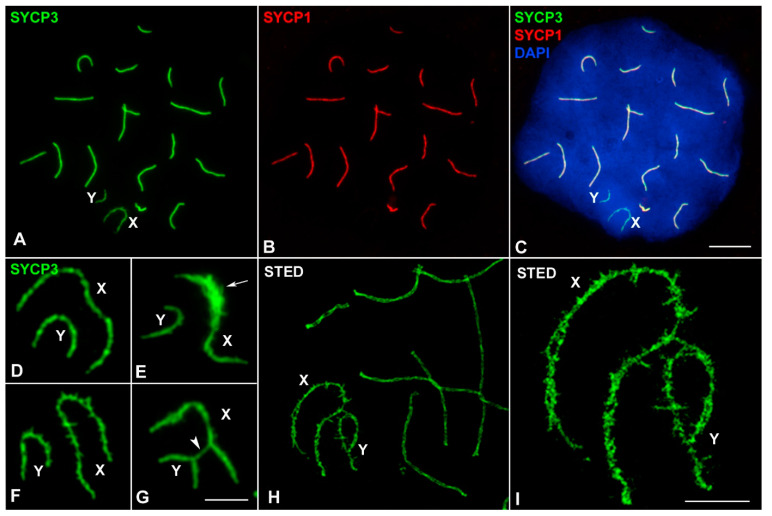
Structural organization of sex chromosomes in prophase-I. (**A**–**C**) Mid pachytene spermatocyte labelled with SYCP3 (green), SYCP1 (red) and DAPI (blue). SYCP1 is absent in the sex chromosomes. A well-defined sex body is observed with DAPI staining. (**D**–**G**) Details of the AEs of the sex chromosomes. (**D**) AEs without obvious modifications. (**E**) The AE of the X chromosome is thickened in an interstitial region (arrow). (**F**) Lateral excrescences are observed in both sex chromosomes. (**G**) Bridges of SYCP3 are occasionally observed between the AEs of sex chromosomes (arrowhead). (**H**) A pachytene spermatocyte observed with super-resolution STED confocal microscopy. The two LEs of autosomes are distinguishable. (**I**) Enlarged image showing sex chromosomes. The AEs of both chromosomes are irregular and show profuse excrescences and splitting into two filaments along their trajectory. Scale bars: 10 µm in (**A**–**C**); 5 µm in (**D**–**I**).

Overall, the spatial and temporal pattern of SYCP3 and γH2AX in the autosomes of *M. mattheyi* is quite similar to that described in *M. musculus* meiosis [14,27]. However, the behavior of sex chromosomes in *M. mattheyi* is rather different. The AEs of the X and Y chromosomes become distinguishable from the rest of chromosomes by late zygotene, and they appear as thinner filaments not involved in synapsis (Figure 2C). At the beginning of pachytene, these AEs appear stretched and separated from each other. Additionally, sex chromosomes are intensely labelled with γH2AX (Figure 2D,E). Although in some early pachytene spermatocytes the γH2AX signal includes both sex chromosomes (Figure 2D) this does not seem to represent a true association. Indeed, in 70.6% of spermatocytes (*n* = 51) at this stage, sex chromosomes lie apart from each other with no contact of either AEs or γH2AX signal (Figure 2E). A stable association is achieved in a later stage, early-mid pachytene (Figure 2F), and is accompanied by morphological changes of the sex chromosomes: their AEs, mainly that of the X chromosome, appear slightly curved, whereas γH2AX is less extended than in previous stages and the outline of the signal surrounding sex chromosomes becomes more regular (Figure 2F). This is followed by the formation of a typical sex body in mid pachytene (Figure 2G and Figure 3C), in which the AEs of both sex chromosomes appear notably bent (often U-shaped) and associated inside a single and well-defined γH2AX signal. This configuration is maintained in late pachytene (Figure 2H) and diplotene (Figure 2I). The labelling of γH2AX during prophase-I suggests that sex chromosomes are subjected to MSCI.

These results indicate that the pairing of sex chromosomes in *M. mattheyi* is delayed if compared to that of other mammals [14]. Moreover, synapsis was never observed between sex chromosomes. Accordingly, the association of SYCP1 protein, the main component of the transverse filaments and the central element of the SC, was not detected in the sex chromosomes (Figure 3A–C). However, structural modifications in the AEs, which are common in many mammals, were observed. SYCP3 labelling revealed the presence of thickenings, excrescences, and lateral projections of the AEs (Figure 3D–F). The use of STED super-resolution microscopy revealed that sex chromosome AEs are highly irregular. We could even observe the presence of splittings and double filaments along the AEs (Figure 3H–I). These modifications are not exclusive to a specific stage, but can be found from mid pachytene to the end of prophase-I. For this reason, the presence of these modifications was not useful for identifying the different sub-stages of pachytene, as it is the case in *M. musculus* [11,14]. Occasionally, we observed that the sex chromosomes can appear connected by SYCP3 bridges (Figure 3G). However, these connections do not represent a true synapsis (they never incorporate SYCP1) but rather the lateral association of the excrescences of their AEs.

### 3.2. DNA Repair/Recombination Dynamics in M. mattheyi

In *M. musculus* DSBs in the Y chromosome usually occur only in the PAR and they are produced at the end of zygotene, thus triggering synapsis between the X and Y chromosomes at this stage [14,21]. The asynaptic nature of sex chromosomes in *M. mattheyi* poses intriguing questions about the dynamics of DSB repair in the sex chromosomes in this species. Thus, in the absence of synapsis, the pattern of DSB production and repair in the sex chromosomes, particularly the Y chromosome, could be altered in comparison to *M. musculus*. To address this question, we analyzed the spatial and temporal location of RPA, RAD51 and MLH1 proteins, which are related with meiotic recombination: RPA is a protein involved in the initial stages of DNA repair, protecting single-stranded DNA regions produced after the induction of DSBs by SPO11; later, RPA is replaced by recombinases RAD51 and DMC1, which promote the invasion of a DNA template and initiate the homologous recombination pathway; finally, MLH1 acts together with MLH3 to transform some of these homologous interactions into crossovers [5,61,62].

**Figure 4:**
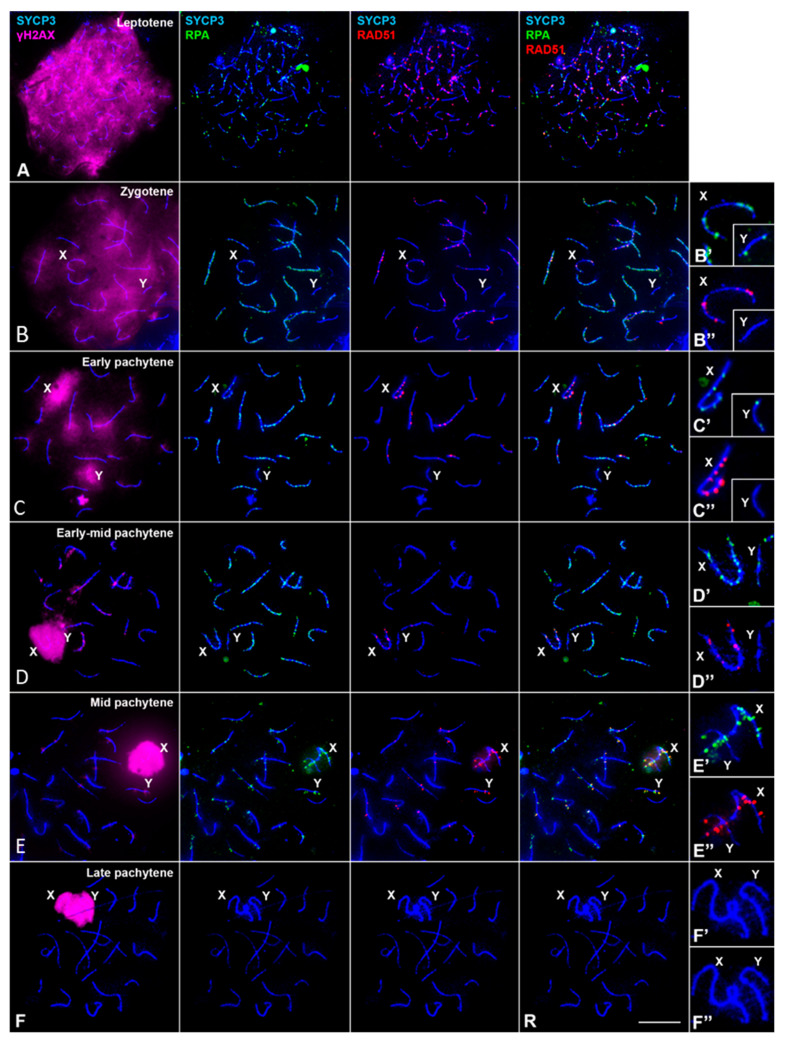
Distribution of RPA and RAD51 during prophase-I in spermatocyte spreads. First column on the left: SYCP3 (blue) and γH2AX (magenta); second column: SYCP3 (blue) and RPA (green); third column: SYCP3 (blue) and RAD51 (red); fourth column: SYCP3 (blue), RPA (green) and RAD51 (red); right column: enlarged details of sex chromosomes for each row. Staging was determined according to SYCP3 and γH2AX labelling patterns, in agreement with Figure 2. (**A**) Leptotene: RPA and RAD51 foci are observed throughout the nucleus associated to forming AEs. (**B**) Zygotene: RPA foci are distributed along synapsed and unsynapsed regions of chromosomes. RAD51 foci are less abundant and mainly localize to unsynapsed regions. Some foci overlap while others do not. Sex chromosomes are distinguishable. The X chromosome shows abundant RPA and RAD51 foci, but the Y chromosome only shows a few RPA foci and rarely accumulates RAD51 foci. (**C**) Early pachytene. The number of RPA and RAD51 foci decreases in the autosomes and the X chromosome. (**D**) Early-mid pachytene. The number of RPA foci is similar to the previous stage, but RAD51 is almost undetectable, except in the X chromosome. (**E**) Mid pachytene. The number of RPA foci further decreases in the autosomes. However, the number of RAD51 foci is conspicuously higher than in the previous stage. Many of these foci appear located out of the SCs in some autosomes. Many RPA foci are observed in the X chromosome, while only a few are detectable in the Y chromosome. Most RPA and RAD51 foci do not colocalize. (**F**) Late Pachytene. RPA and RAD512 are not detected in either the autosomes or the sex chromosomes. Scale bar: 10 µm.

The distribution and dynamics of RPA in *M. mattheyi* throughout meiosis is similar to those previously described in *M. musculus* and humans [63,64]. A large number of small RPA foci appear at leptotene and zygotene located over the AEs or the SC of autosomes, thus overlapping with the SYCP3 signal (Figure 4A,B–B″). At zygotene these foci accumulate largely on those chromosomal regions that have completed synapsis. During pachytene, the number of RPA foci progressively decreases (Figure 4C–E″) and by late pachytene they are no longer detected (Figure 4F–F″). Moreover, RPA can be observed associated to both the X and the Y chromosomes, both before (Figure 4C–C″) and after sex chromosome pairing (Figure 4D–D″). However, we found that while the X chromosome regularly accumulates RPA foci, the Y chromosome accumulates this protein in a reduced proportion of cells: 56.3% (*n* = 80) in late zygotene, 56.0% (*n* = 75) in early pachytene and 43.0% (*n* = 200) in mid pachytene. When present, the number of RPA foci on the Y chromosome is limited, ranging from 1 to 5 (mean values: 1.66, 1.78 and 1.69 in zygotene, early pachytene and mid pachytene respectively). This indicates that the dynamics of RPA in this chromosome is clearly different from the autosomes and the X chromosome.

We next analyzed the location of recombinases RAD51 and DMC1. Unfortunately, the use of up to three different antibodies against DMC1 did not produce any specific labelling. Therefore, we focused on the analysis of RAD51 (Figure 4). From leptotene to mid pachytene, RAD51 is located in the nucleus as small foci associated to AEs/SCs. The dynamics of RAD51 are quite similar to that of RPA, although there are relevant differences. First, at zygotene RAD51 and RPA mainly concentrates in unsynapsed and synapsed regions, respectively (Figure 4B). Indeed, although colocalization is observed, most foci do not overlap. Second, RAD51 foci are barely detectable at the early-mid pachytene stage (Figure 4D), but the number of foci increases during mid pachytene (Figure 4E). These foci seem larger than those observed in early pachytene spermatocytes and often project laterally out of the SC, both on the autosomes and on the X chromosome. Finally, while RAD51 is regularly observed in the X chromosome, the proportion of spermatocytes with RAD51 on the Y chromosome is lower compared to RPA. Thus, only 24.43% of zygotene (*n* = 131), 34.26% of early pachytene (*n* = 108) and 10.62% of mid pachytene spermatocytes (*n* = 339) have RAD51 foci on the Y chromosome. When present, a single focus of RAD51 is observed. Altogether, these results indicate that most spermatocytes do not exhibit RAD51 on the Y chromosome. Moreover, after double localization with RPA, we found that RAD51 foci were only located in the Y chromosome when RPA foci were also present, although, as observed in autosomes, both kinds of foci rarely colocalize (Figure 4E).

**Figure 5:**
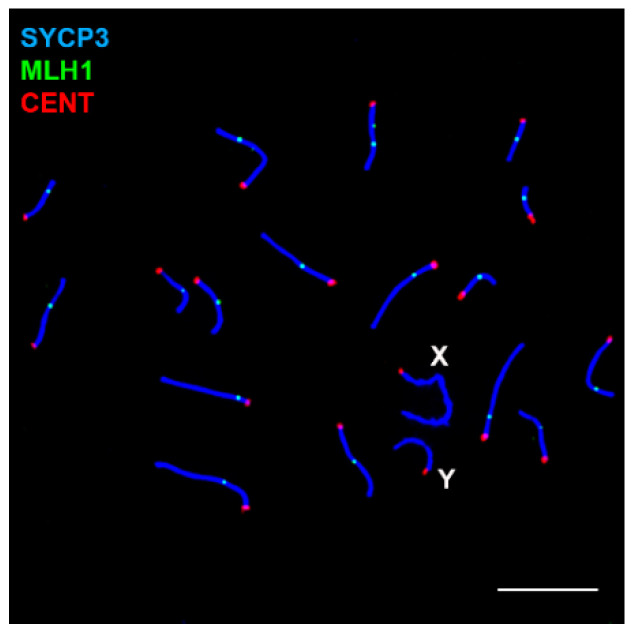
Distribution of MLH1 in a pachytene spermatocyte. SYCP3 (blue), MLH1 (**green**) and centromeres (**red**). All autosomes show at least one MLH1 focus. Sex chromosomes do not have any focus. Scale bar: 10 µm.

To conclude the analysis of recombination markers, we studied the localization of MLH1, which is involved in the resolution of recombination intermediates and in the formation of crossovers. This protein is detectable in mid and late pachytene spermatocytes as discrete foci over the SCs of autosomes (Figure 5). Each of the 17 autosomal bivalents shows at least one (occasionally two) focus, indicating the formation of at least one chiasma per bivalent. As expected, sex chromosomes do not have any MLH1 signal on their AEs at any time.

### 3.3. Sex Chromosome Segregation

**Figure 6:**
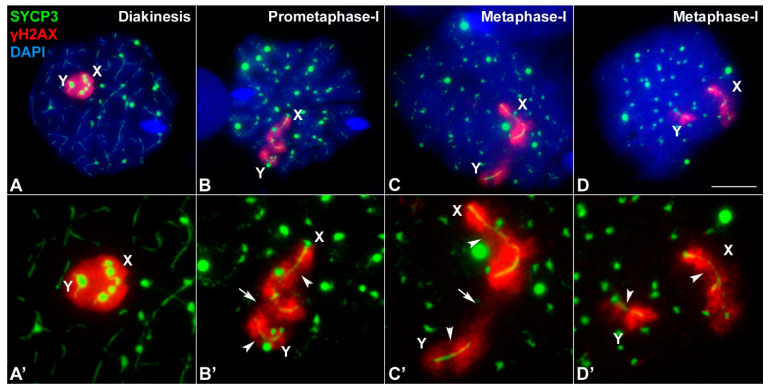
Association of sex chromosomes during late stages of first meiotic division. Spread spermatocytes labelled with antibodies against SYCP3 (green) and γH2AX (red) and counterstained with DAPI (blue). (**A′**–**D′**) shows enlarged images of the sex chromosomes presented in (**A**–**D**). (**A**,**A′**) Diakinesis: SYCP3 appears as thin lines along each bivalent. Some protein aggregates are observed, particularly in the sex chromosomes. γH2AX signal covers the sex chromosomes, which are closely associated. (**B**,**B′**) Prometaphase-I. SYCP3 is observed as a discontinuous line inside each bivalent, and abundant SYCP3 aggregates decorate the nucleus. SYCP3 is also observed running inside the X and Y chromosome. The X chromosome shows a conspicuous bending, adopting a hook-like shape. The γH2AX signal in the sex chromosomes becomes discernible for each chromosome, although both chromosomes are still closely associated (arrow). Some regions along sex chromosomes have a weaker labelling with γH2AX (arrowheads). (**C**) Metaphase-I. SYCP3 signal becomes weaker inside the autosomes and preferentially accumulates in the centromeric regions. Sex chromosomes are clearly more separated than in previous stages, but there is a γH2AX bridge connecting them (arrow). The X chromosome still has a hook-like configuration. (**D**) Metaphase-I. SYCP3 signal is barely detectable inside the autosomes and mostly accumulates in the centromeric regions. Sex chromosomes have apparently lost their contact. γH2AX weaker regions are still differentiated in the sex chromosomes (arrowheads). Scale bar: 10 µm.

The asynaptic and achiasmatic nature of the sex chromosomes observed in *M. mattheyi* raises the question of how these chromosomes are able to properly segregate during the first meiotic division. In spread spermatocytes, we could observe that at diakinesis sex chromosomes remain closely associated within the sex body. SYCP3 appears as a well-defined filament with large spherical accumulations within each chromosome (Figure 6A). At prometaphase-I, the labelling inside the chromosomes is patent while the spherical accumulations disappear gradually (Figure 6B). Strikingly, we observed that sex chromosomes usually adopt a particular configuration: the X chromosome appears bent at an interstitial region, adopting a shape similar to a hook; the distal end of the Y chromosome appears facing this region of the X chromosome. This configuration is also observed in later stages (metaphase-I) (Figure 6C,D).

Contrary to *M. musculus*, in which γH2AX labelling in the sex chromosomes is usually lost during prometaphase-I or metaphase-I (Figure S1), we found that in *M. mattheyi* γH2AX remains into later stages, allowing a detailed observation of the chromatin organization of these chromosomes. At diakinesis (Figure 6A), sex chromosomes present the configuration of previous stages, forming a single and compact chromatin body intensely labelled with γH2AX. However, at prometaphase-I the γH2AX signals of X and Y chromosomes become discernible from each other, although they remain in contact (Figure 6B). At metaphase-I, γH2AX signal further changes as some interstitial regions along both chromosomes are devoid of labelling (Figure 6C,D). In some spermatocytes the chromatin of X and Y chromosomes remain in contact through the γH2AX signal (Figure 6C), while in others, putatively in more advanced stages, this connection is apparently lost (Figure 6D).

**Figure 7:**
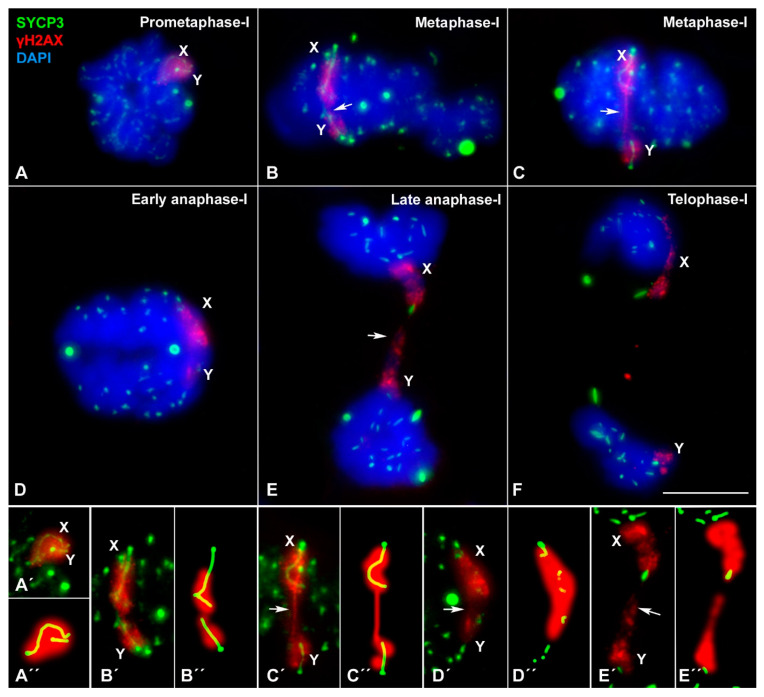
Segregation of sex chromosomes during first meiotic division. Squashed spermatocytes labelled with antibodies against SYCP3 (green) and γH2AX (red) and counterstained with DAPI (blue). Lower row shows enlarged images of the sex chromosomes presented in (**A**–**E**). (**A**,**A′**) Prometaphase-I. SYCP3 signal is observed in the autosomes, while γH2AX marks sex chromosomes, which are tightly associated at the nucleus periphery. (**B**,**B′**) Metaphase-I. Bivalents appear aligned at the metaphase plate. SYCP3 in autosomes accumulates especially in centromeric regions and in some large scattered foci. Sex chromosomes are oriented to opposite poles and appear associated and labelled with γH2AX, which is continuous between both chromosomes (arrow). The hook-like configuration of the X chromosome is discernible. (**C**,**C′**) Metaphase-I. Autosomal bivalents are aligned at the cell equator, while the sex chromosomes appear further apart. A γH2AX filament bridges from the X to the Y chromosome (arrow). (**D**,**D′**) Early anaphase-I. Autosomes start migration. SYCP3 is observed as aggregates in the centromeric regions and as large accumulation in the cytoplasm. Sex chromosomes initiate their segregation, but a γH2AX contact is still observed (arrow). (**E**,**E′**) Late anaphase-I. Two chromatin masses appear clearly separated. A chromatin connection between these two chromosome groups is observed (arrow), most probably representing sex chromosomes. (**F**) Telophase-I. Chromosomes reach the cell poles. A γH2AX signal is detected in each pole, but without connection between them. (**A″**–**E″**) shows enlarged images of the sex chromosomes presented in (**A′**–**E′**). Scale bar: 10 µm.

These results indicate that sex chromosomes maintain their association during the first meiotic division, but this association seems to be lost at some point during metaphase-I. In order to elucidate whether this sequence of interactions is related to the orientation of the sex chromosomes in the metaphase-I plate, we processed the spermatocytes using a squash protocol that maintains the 3D organization of cells and has been previously used to address this problem in other species [43,45,46,60,65]. In prometaphase-I, sex chromosomes, which are clearly distinguishable by their γH2AX labelling, remain tightly associated (Figure 7A). At metaphase-I, once bivalents have oriented in the meiotic spindle, sex chromosomes appear oriented towards opposite cell poles. The X chromosome still shows a bending towards where one end of the Y chromosome is oriented (Figure 7B). No physical connection of the internal axial structures of sex chromosomes, like a SYCP3 filament, was observed. Instead, the two sex chromosomes maintain their contact by a chromatin association that is labelled with γH2AX (Figure 7B). In some cases, the segregation starts prematurely for sex chromosomes (Figure 7C) but a connection persists through the γH2AX filament. The X chromosome appears bent in its distal third (hook-like shape) while the Y chromosome appears fully stretched and oriented towards the X. The SYCP3 signal is still visible at the interchromatid domain of the autosomes, indicating that anaphase-I has not started. During anaphase-I, sex chromosomes migrate to opposite poles (Figure 7D). We noticed the presence of a chromatin connection labelled with γH2AX, but not with SYCP3 (Figure 7E). Finally, the chromatin bridge disappears at telophase-I (Figure 7F).

## 4. Discussion

The evolution of sex chromosomes is a very active field in evolutionary biology. Sequencing of these chromosomes has provided a deep understanding of the events in the past that have conditioned their organization and function [66,67]. Although this topic has traditionally been considered from a genetic perspective, recent works have demonstrated that meiosis is an important factor to understand the evolutionary dynamics of sex chromosomes [55,68,69,70,71].

The meiotic behavior of sex chromosomes in the laboratory and wild house mouse (*M. musculus*) has been extensively studied for decades [11,12,13,14,72]. This species served to explore the particularities of sex chromosome synapsis, recombination, inactivation and segregation [19,22,73,74,75,76,77]. These studies, which include genetically manipulated models, have revealed that the alteration of sex chromosome behavior has dramatic consequences for meiosis progression and fertility, thus impeding our understanding of how these changes can be transmitted in an evolutionary context. The study of meiosis in *M. mattheyi* presented here offers new clues to understand how the alterations of sex chromosome pairing, synapsis and recombination, which cause severe meiosis impairment in the house mouse, can be overcome in closely related species, allowing sex chromosomes to evolve to complete differentiation.

### 4.1. Origin of the Asynaptic Condition of Sex Chromosomes in M. mattheyi

Although previous works suggested that synapsis should not occur for sex chromosomes of *M. mattheyi* [54,55], our results provide conclusive cytological evidence. Thus, we have found that: (i) the sex chromosomes never align side by side, (ii) there is no lateral contact between their AEs and (iii) SYCP1 protein is not incorporated to these chromosomes. We occasionally observed lateral contacts between sex chromosomes, but this is mostly due to the presence of SYCP3 excrescences along their AEs. In addition, according to our results on MLH1 localization, sex chromosomes are also achiasmate.

Asynaptic sex chromosomes are rare in mammals, but are common in some groups of rodents such as gerbils and voles [29,30,31,32,33,45,46,78], and are a distinctive feature in marsupials [79,80]. Our work confirms that this feature is also shared by pygmy mouse species of the subgenus *Nannomys*. The absence of synapsis is generally accepted to derive from a complete differentiation of sex chromosomes, involving the loss of the PAR, at least, in the Y chromosome [30,37]. This is a consequence of the genetic isolation of the Y chromosome once recombination with the X chromosome is reduced or abolished. In this scenario, the Y chromosome is subjected to both random drift and selection forces that produce its genetic erosion, which ultimately leads to a complete differentiation from the X chromosome [81,82,83]. This phenomenon would have occurred several times in the evolution of mammals. In the case of marsupials, the homology between the X and Y chromosomes was completely lost before the radiation of this group and it has not been restored. However, in eutherian mammals the homology was probably lost and then restored again by the translocation of an autosomal segment to both sex chromosomes, giving rise to the appearance of the current PAR region about 120 million years ago [66]. Subsequently, some groups lost the PAR again. This process is known as the addition-attrition cycle of sex chromosome evolution [84]. This would be the case of gerbils and vole species with asynaptic sex chromosomes, in which this condition is clearly derived [30,45,46,85]. Likewise, since the asynaptic condition of sex chromosomes is not present in any other *Mus* species outside the *Nannomys* subgenus, it could also be considered a derived feature of this group. Loss of the PAR is the most plausible explanation for the asynaptic condition of sex chromosomes in *M. mattheyi* (although additional explanations are possible, see below). However, this may not be a terminal situation for sex chromosomes in mice within the *Nannomys* subgenus. This should be placed in the context of the high karyotypic variability described in this group [52]. Indeed, sex chromosomes of *Nannomys* are regularly engaged in new translocation events with autosomes, as described in *M. minutoides*. In this species the completely differentiated X and Y chromosomes have been translocated to chromosome 1, generating a neo-XY chromosome pair with a large neo-PAR, which now combines the coexistence of a chiasma in the neo-PAR and the preservation of an ancestral achiasmate association in the differentiated segments [55,86]. Interestingly, among the gerbils, voles, and marsupials, there are also some species that present sex-autosome translocations [87,88,89]. The recurrent association of two rare events, sex chromosome asynapsis and sex-autosome translocation, may suggest that the asynaptic condition could support a higher rate of fixation of sex-autosome translocations to restore a neo-PAR to ensure a correct (better?) segregation of the X and the Y. This process mirrors the addition-attrition model of sex chromosome evolution [84]. Nevertheless, within these four groups, many species with asynaptic sex chromosomes (not translocated to autosomes) persist and do not seem to have a higher rate of aneuploidy, although statistical comparisons are lacking.

### 4.2. DNA Damage and Repair in the Absence of Synapsis

Loss of synapsis between sex chromosomes challenges two critical aspects of their meiotic behavior: DNA repair and pairing. This is especially intriguing, taking into account that hampering either pairing or recombination between sex chromosomes in *M. musculus* laboratory strains causes meiosis impairment, severe fertility defects, sex chromosome mis-segregation and aneuploidy [73,90]. Therefore, the absence of synapsis and recombination requires the emergence of alternative mechanisms to ensure meiosis success.

As indicated above, in most mammals, sex chromosome synapsis is dependent on programmed DNA damage that is subsequently repaired through homologous recombination. In *M. musculus* the induction of DSBs in the PAR is extremely regulated, relaying on the action of the specific SPO11α isoform [21] and the accumulation of many recombination factors, forming the RMMAI complex [23,24,25]. Furthermore, the accumulation of these proteins is accompanied by a structural modification of the AEs and lengthening of chromatin loops [23]. Soon after DSB induction, RAD51 and DMC1 loads to the PAR, triggering synapsis between sex chromosomes [14]. According to their critical role, loss of SPO11α or ANKRD31 leads to failure of sex chromosome synapsis and cause meiotic arrest at metaphase-I [24,25,91].

In *M. mattheyi* we did not observe the structural modifications encompassed by the PAR in *M. musculus*, even under super-resolution fluorescence microscopy [23]. However, as regards the presence of an intense γH2AX signal, both the X and the Y chromosomes seem to accumulate DNA damage. It is not clear if the induction of this damage in *M. mattheyi* would be regulated by the same mechanisms acting in *M. musculus*, but at least two differences are relevant about the subsequent repair of DNA on the Y chromosome. First, induction of damage is not followed by the expected accumulation of DNA repair proteins. Up to 46% and 70% of spermatocytes do not incorporate RPA or RAD51 on the Y chromosome, respectively. The absence of these proteins suggests that a strikingly high proportion of spermatocytes may repair DNA in the Y chromosome using a pathway different from homologous recombination. Second, the noticeable difference in the Y chromosome between the proportion of RPA foci compared to that of RAD51 suggests that upon DNA resection, this damage would not be repaired by the canonical homologous recombination pathway using the sister chromatid as template, as previously suggested [22,26,27,28]. Instead, repair could be achieved by alternative mechanisms that do not involve recombinases, the single strand-annealing pathway, for instance. Although this possibility may seem unlikely, previous studies in *Caenorhabditis elegans* have suggested that single strand-annealing can indeed be involved in the repair of the single X chromosome in male meiosis [92].

These striking features of DNA repair on the Y chromosome open interesting possibilities to understand the cessation of recombination between the X and Y chromosomes. Although the deletion of the PAR is the most plausible cause to explain the asynaptic and achiasmate nature of sex chromosomes in *M. mattheyi*, slight changes in the regulation of DNA repair, or the diversion of this repair to non-recombinogenic pathways, could have a similar effect. In both cases, the homologous recognition of X and the Y chromosomes would be hampered and thus recombination would be abrogated. Under this scenario, it is possible to speculate that loss-of-function mutations in genes like SPO11α or AKRD31, slight changes in the microsatellites that drive DSB production in the PAR [23], or modifications in the regulation of DNA resection could lead to an effective genetic isolation of the PAR in the Y chromosome. Then, the effects of random drift and selection could lead to the complete differentiation of the Y chromosome.

### 4.3. Sex Chromosome Pairing in the Absence of Homologous Recombination

Albeit sex chromosomes do not synapse, pairing between them becomes necessary for ensuring proper segregation. As observed in other species with asynaptic sex chromosomes [16,31,37,38] our results show that pairing of the sex chromosomes in *M. mattheyi* is delayed. How do these homology-independent pairing mechanisms work? Sex chromosomes could just move on the nuclear envelope as a consequence of the general movements of chromosomes during pachytene [93,94] and they could encounter just by chance. If this would be the case, then we should expect that sex chromosomes pair at any time during pachytene. However, we observed that pairing is coincident with precise morphological changes of AEs and the γH2AX signal in both autosomes and sex chromosomes. Therefore, pairing could rely on more precisely regulated events. Interestingly, sex chromosome pairing is not abrogated in SPO11α or ANKRD31 knockout *M. musculus* models [21,24,25]. This strongly suggests that sex chromosome pairing is functionally distinguishable from synapsis and it could be independent of homologous recombination. Thus, it is tempting to speculate that alternative, and evolutionary conserved, mechanisms are indeed responsible of sex chromosome pairing. It was proposed that the cytoskeleton could serve as a polarizing structure that favors sex chromosome encounter [38], but direct proof is still lacking. In any case, upon pairing, sex chromosomes remain associated side by side and a typical sex body is formed, as revealed by the condensation of the chromatin and the intense accumulation of γH2AX. This seems sufficient to maintain sex chromosomes associated during the rest of prophase-I.

### 4.4. An Unreported Mechanism of Sex Chromosome Association at Metaphase-I

Different mechanisms have been suggested for ensuring the segregation of achiasmate sex chromosomes. In marsupials, the persistence of the dense plate up to metaphase-I was proposed to act for that purpose [43]. The dense plate incorporates some components of the SC, most relevantly SYCP3. This protein was subsequently proposed to also act also in the segregation of sex chromosomes in gerbils and voles [45,46]. On these grounds, it was expected that in *M. mattheyi* sex chromosomes could display a similar mechanism. However, we have not found any signs of SYCP3 involvement in this association. Although the distal end of the Y chromosome seems to be oriented to an interstitial region of the X chromosome, we have not observed a physical contact between these SYCP3 labelled regions. Instead, the sex chromosomes seem to remain associated just through a chromatin contact. The association through heterochromatin regions between asynaptic sex chromosomes has already been suggested for the species *Psammomys obesus* [29,33] and for voles with large distal heterochromatic blocks [34,36]. However, *M. mattheyi* does not exhibit such heterochromatin regions.

Therefore, the association of sex chromosomes through chromatin interactions in *M. mattheyi* seems to represent just a remnant of the sex body formation during pachytene, which entails the condensation of chromatin and the incorporation of a number of epigenetic modifications [14,17,76,95,96]. This mode of association seems not to be sufficient in *M. musculus* models with defects in either synapsis or recombination of sex chromosomes [21,24,25]. In those cases sex chromosomes separate prematurely and trigger apoptosis at metaphase-I by the activation of the spindle assembly checkpoint [91,97]. However, a relevant difference in *M. mattheyi* is the persistence of γH2AX in the sex chromosomes through the first meiotic division. This histone modification has been observed to persist in the achiasmate sex chromosomes of other mammalian species. This is the case of the Mongolian gerbil *Meriones unguiculatus* and the Mediterranean pine vole *Microtus duodecimcostatus* [45,46]. The persistence of γH2AX, and putatively other proteins involved in the formation of the sex body, could represent a reliable mechanism to ensure achiasmate sex chromosome association and segregation. Thus, by slightly tuning the regulation of H2AX dephosphorylation and/or turnover at the end of prophase-I, sex chromosomes that have achieved a complete differentiation could be efficiently transmitted to offspring. Strikingly, this same mode of association has been retained in the heterologous segments of sex chromosomes of *M. minutoides* after their translocation to an autosome [55]. This reveals that a dual mode of segregation can be present after restoration of homology between sex chromosomes. Interestingly, in some populations of *M. minutoides* chromosomal fissions seem to have restored the original (unfused) condition of sex chromosomes [98]. The preservation of the dual mechanism of sex chromosome association could have contributed to the proper transmission of sex chromosomes under these circumstances.

## 5. Conclusions

The results presented here offer new clues about the behavior of sex chromosomes during meiosis. The close evolutionary proximity of *M. mattheyi* with *M. musculus* offers the possibility to undertake genomic comparisons that could reveal slight but significant differences with consequences for the outcome of meiosis. It is particularly interesting that small changes in the regulation of DNA repair or epigenetic modifications of sex chromosomes can have a profound impact on the subsequent transmission and evolution of these chromosomes, particularly the Y chromosome. Thus, the final stages of sex chromosome differentiation could be achieved more easily than anticipated, provided that accurate mechanisms for pairing and segregation are working. Obviously, meiosis emerges as an important factor to be considered in the study of sex chromosome evolution.

## Data Availability

Not applicable.

## Supplementary Materials

The following are available online at https://www.mdpi.com/article/10.3390/genes12091434/s1, Figure S1: γH2AX labeling of sex chromosomes during first meiotic division in *M. musculus*.
