# Supplementary material for: Meiotic Behavior of Achiasmate Sex Chromosomes in the African Pygmy Mouse Mus mattheyi Offers New Insights into the Evolution of Sex Chromosome Pairing and Segregation in Mammals
Source: Genes (Basel). 2021 Sep 17;12(9):1434. doi: 10.3390/genes12091434 (PMC8471055; doi:10.3390/genes12091434)
Supplement: Supplementary file 1 [file genes-12-01434-s001.zip › genes-1373675-supplementary.pdf]

Supplementary Figure S1

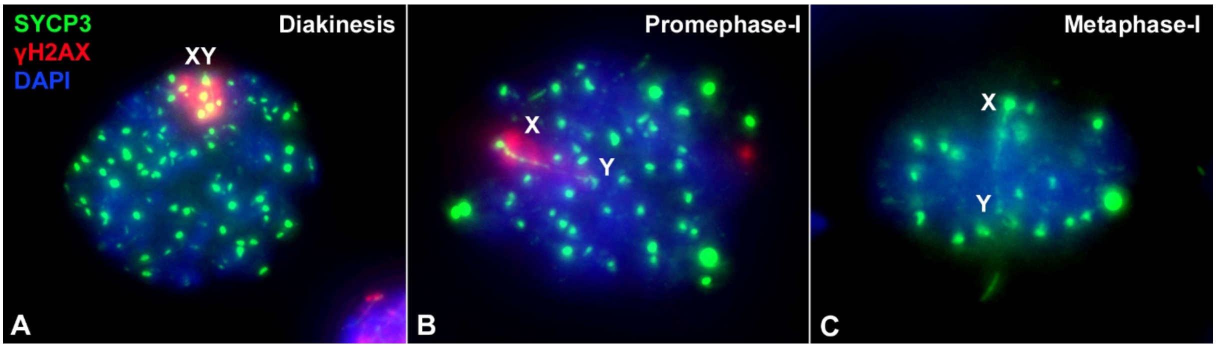

Supplementary Figure S1. γH2AX labeling of sex chromosomes during first meiotic division in *M. musculus*. Squashed spermatocytes labelled with antibodies against SYCP3 (green) and γH2AX (magenta) and counterstained with DAPI (blue). Sex chromosomes are labelled with γH2AX in diakinesis (**A**), but only a weak labelling is conserved in prometaphase-I (**B**), and no γH2AX is detected in metaphase-I (**C**). Scale bar: 10 μm.
